# Activation of Brain Somatostatin Signaling Suppresses CRF Receptor-Mediated Stress Response

**DOI:** 10.3389/fnins.2017.00231

**Published:** 2017-04-25

**Authors:** Andreas Stengel, Yvette F. Taché

**Affiliations:** ^1^Division of Psychosomatic Medicine, Charité Center for Internal Medicine and Dermatology, Charité-Universitätsmedizin BerlinBerlin, Germany; ^2^Vatche and Tamar Manoukian Digestive Diseases Division, CURE Digestive Diseases Research Center, G Oppenheimer Center for Neurobiology of Stress and Resilience, Department of Medicine, University of California, Los AngelesLos Angeles, CA, USA; ^3^VA Greater Los Angeles Health Care SystemLos Angeles, CA, USA

**Keywords:** brain-gut axis, food intake, gastrointestinal functions, HPA, hypothalamus, stress

## Abstract

Corticotropin-releasing factor (CRF) is the hallmark brain peptide triggering the response to stress and mediates—in addition to the stimulation of the hypothalamus-pituitary-adrenal (HPA) axis—other hormonal, behavioral, autonomic and visceral components. Earlier reports indicate that somatostatin-28 injected intracerebroventricularly counteracts the acute stress-induced ACTH and catecholamine release. Mounting evidence now supports that activation of brain somatostatin signaling exerts a broader anti-stress effect by blunting the endocrine, autonomic, behavioral (with a focus on food intake) and visceral gastrointestinal motor responses through the involvement of distinct somatostatin receptor subtypes.

## Introduction

The past years have witnessed major advances in our understanding of the underlying mechanisms involved in the bodily response to stress (Chrousos and Zoumakis, 2017). Namely, the corticotropin-releasing factor (CRF) systems in the brain play a major role in coordinating an array of stress-related behavioral, endocrine, autonomic and visceral changes as well as the stress recovery through activation of distinct CRF receptor subtypes (Bale and Vale, 2004; Taché and Million, 2015; Henckens et al., 2016). This was established by monitoring alterations of CRF systems occurring during stress exposure in specific brain nuclei and the impact of pharmacological or targeted genetic manipulations of CRF ligands and/or receptors on the stress response (Suda et al., 2004; Chen et al., 2014; Rivier and Rivier, 2014; Taché and Million, 2015; Henckens et al., 2016). Simultaneously, other brain pathways are recruited by stress that exert stress-relieving effects (Bali et al., 2014). Among them, the activation of brain neuropeptide Y_1_ and oxytocin receptors have been implicated in stress adaptation processes as recently reviewed (Zheng et al., 2010; Reichmann and Holzer, 2016). Earlier reports by Brown et al. showed that the injection of somatostatin-28 into the lateral brain ventricle blocked acute stressors-induced rise of ACTH and epinephrine plasma levels in rats (Brown et al., 1984). The present review will summarize mounting evidence indicating that activation of brain somatostatin signaling at different brain sites exerts an anti-stress action that extends to several components of the stress response through distinct somatostatin receptor subtypes.

### Brain corticotropin releasing factor signaling

In 1950, Harris and colleagues reported that different stressors stimulate the release of adrenocorticotropic hormone (ACTH) *via* a yet unknown hypothalamic factor (de Groot and Harris, 1950; Harris, 1950). Five years later, Guillemin and coworkers purified a factor able to stimulate pituitary ACTH secretion using a large sample of bovine hypothalami (Guillemin and Rosenberg, 1955; Saffran et al., 1955). This factor was named CRF (Guillemin and Rosenberg, 1955; Saffran et al., 1955). However, it took another 26 years to identify and sequence the 41-amino acid peptide that plays a pivotal role in the stress-related pituitary release of ACTH and β-endorphin (Vale et al., 1981; Bale and Chen, 2012). Besides acting as a secretagogue of the hypothalamus-pituitary-adrenal (HPA) axis, CRF was subsequently implicated in stress-related alterations of autonomic (Yang et al., 2010; Bardgett et al., 2014), visceral (Taché and Million, 2015), behavioral (Bale and Vale, 2004), and also immune (Gravanis and Margioris, 2005) responses.

Following the characterization of CRF, additional structurally related members of the CRF peptide family were identified, namely urocortin 1 (Ucn 1, 40 amino acids, 45% sequence homology with rat/human CRF) (Vaughan et al., 1995), Ucn 2 (39 amino acids, 34% sequence homology with rat/human CRF) (Reyes et al., 2001) and Ucn 3 (38 amino acids, 26% sequence homology with rat/human CRF) (Lewis et al., 2001). These peptides are encoded by distinct genes highly conserved across mammalian and non-mammalian species (Lovejoy and de Lannoy, 2013).

Mapping studies identified prominent CRF expression in the cerebral cortex, amygdala, hippocampus and the Barrington's nucleus in rodents (Wang et al., 2011). Likewise, urocortins display broad distribution although there is little overlap with that of CRF. Ucn 1 immunoreactivity is mainly expressed in the Edinger-Westphal nucleus (Bittencourt et al., 1999), while Ucn 2 mRNA (due to the lack of a specific antibody) has been detected in the supraoptic nucleus, the paraventricular and arcuate nucleus of the hypothalamus, the locus coeruleus, several cranial nerve motor nuclei and the ventral horn of the spinal cord (Reyes et al., 2001; Mano-Otagiri and Shibasaki, 2004). Lastly, Ucn 3 mRNA and peptide expression have been identified in the amygdala, lateral septum, ventromedial hypothalamus and paraventricular nucleus of the hypothalamus, basomedial nucleus of the stria terminalis, dorsal raphe nucleus and the area postrema (Lewis et al., 2001; Li et al., 2002; Mano-Otagiri and Shibasaki, 2004; Venihaki et al., 2004).

The various biological effects of CRF and Ucns are mediated by binding to and activating two distinct seven-transmembrane domain (TMD) G-protein-coupled receptor subtypes, CRF_1_ and/or CRF_2_ that belong to the B1 subfamily (Perrin and Vale, 1999). CRF ligands display distinct affinity to CRF receptors with CRF binding preferentially to CRF_1_ and with lesser affinity to CRF_2_ (Hillhouse and Grammatopoulos, 2006), while Ucn 1 displays equal high affinity to both CRF_1_ and CRF_2_ and Ucn 2 and 3 are selective CRF_2_ agonists (Grace et al., 2007). Both CRF receptors are encoded by distinct genes which exhibit diverse alternative pre-mRNA splicing patterns resulting in multiple variants derived from partial or total exon deletions or insertions (Grammatopoulos et al., 1999; Pisarchik and Slominski, 2001; Wu et al., 2007, 2011; Zmijewski and Slominski, 2010; Grammatopoulos, 2012; Yuan et al., 2012, 2016). With regard to the nine human CRF_1_ variants, CRF_1a−i_, described, CRF_1a_ being the main wild type functional receptor while the other isoforms may modulate CRF signaling (Zmijewski and Slominski, 2010; Wu et al., 2011). For the CRF_2_, three functionally active splice variants, CRF_2a−c_, have been described in humans (Hillhouse and Grammatopoulos, 2006).

In line with the widespread expression of CRF ligands, CRF_1_ and CRF_2_ are also widely distributed in the rodent brain (Van Pett et al., 2000; Justice et al., 2008). CRF_1_ is prominently expressed in the forebrain including the isocortex throughout cortical layers II-VI, hippocampal formation at the CA1 level, basal ganglia within the globus pallidus and striatum, sensory systems and the amygdala (Justice et al., 2008; Kuhne et al., 2012), while basal levels are low in the hypothalamus (Bonaz and Rivest, 1998) and spinal cord (Kuhne et al., 2012). Moreover, CRF_1_ was also detected in all segments of the mouse spinal cord throughout laminae II-V (Korosi et al., 2007). The CRF_2_ shows a wide distribution in the brain, most notably in the amygdala, lateral septum, supraoptic nucleus, ventromedial hypothalamus, dorsal raphe nuclei, area postrema, the nucleus of the solitary tract and the spinal cord (Bittencourt and Sawchenko, 2000; Korosi et al., 2007; Lukkes et al., 2011).

### Brain somatostatin signaling

Somatostatin-14 was isolated in 1973 from ovine hypothalami and shown to inhibit growth hormone secretion *in vitro* (Brazeau et al., 1973). Seven years later, the N-terminally extended form, somatostatin-28, generated by differential posttranslational processing from a common precursor molecule, was identified (Pradayrol et al., 1980). The somatostatinergic system also encompasses cortistatin, an evolutionary-related peptide that shares high structural and functional similarity to somatostatin although derived from a distinct gene (de Lecea et al., 1996; Gahete et al., 2008). In the rat brain, the pre-pro-hormone gives rise to cortistatin-14 and -29, while in humans, it leads to a 17-amino acid peptide (Hannon et al., 2002a).

Besides the initially described expression site in the hypothalamus, somatostatin is widely distributed in the rodent brain with dense expression in the cortex, amygdala, limbic and sensory system, periaqueductal central gray and paraventricular, ventromedial and arcuate hypothalamic nuclei (Finley et al., 1981; Johansson et al., 1984; Moga and Gray, 1985; Viollet et al., 2008).

Somatostatin-14 and somatostatin-28 bind to five receptor subtypes (sst_1−5_), all belonging to G-protein-coupled TMD receptors encoded by different non-allelic and intronless genes (Theodoropoulou and Stalla, 2013). Different functionally active isoforms have been described for the sst_2_ and sst_5_. The full length sst_2a_ and the truncated sst_2b_ differ only in the length and composition of their C-terminal domains (Cole and Schindler, 2000). The truncated sst_5_ variants differ by their shorter C-terminal tails and display less than seven TMD which vary between species and have been named accordingly (rat sst_5_TMD1; mouse sst_5_TMD1, sst_5_TMD2 and sst_5_TMD4; pig sst_5_TMD3 and sst_5_TMD6; human sst_5_TMD4 and sst_5_TMD5) (Duran-Prado et al., 2009, 2012; Cordoba-Chacon et al., 2010).

In line with the mapping of the ligand, the sst receptors are widely expressed in the rodent brain with the following regional pattern: all layers of the cerebral cortex (sst_1_, sst_2a/b_, sst_3_, sst_4_), bed nucleus of the stria terminalis (sst_1_, sst_2a, b_, sst_4_), hippocampus (sst_1_, sst_2a, b_, sst_3_, sst_4_), basolateral amygdaloid nucleus (sst_1_, sst_2a/b_, sst_3_, sst_4_), medial amygdaloid nucleus (sst_1_, sst_2_, sst_3_), ventromedial hypothalamic nucleus (sst_1_, sst_2_, sst_3_), dorsomedial hypothalamic nucleus (sst_1_, sst_3_), paraventricular nucleus (sst_2a_, sst_3_) and arcuate nucleus of the hypothalamus (sst_1_, sst_2a_, sst_3_, sst_4_), substantia nigra (sst_1_, sst_2a/b_, sst_3_), dorsal raphe nucleus (sst_1_, sst_2_, sst_3_), locus coeruleus (sst_2_, sst_3_), granular layer of the cerebellum (sst_1_, sst_2b_, sst_3_, sst_4_, sst_5_), dorsal motor nucleus of the vagus nerve (sst_2a, b_, sst_4_, sst_5_) and nucleus of the solitary tract (sst_1_, sst_2_, sst_3_) (Schindler and Humphrey, 1999; Fehlmann et al., 2000; Schulz et al., 2000; Hannon et al., 2002b; Videau et al., 2003; Spary et al., 2008; Kumar, 2012). With regards to the truncated sst_5_ variants, they show a distinct distribution with a high abundance of full length sst_5_ in mouse hypothalamus and cerebellum followed by sst_5_TMD2 and sst_5_TMD1, whereas sst_5_TMD4 is not detectable (Hannon et al., 2002b; Cordoba-Chacon et al., 2010). By contrast, in the mouse cortex full length sst_5_ is absent, while all truncated variants are expressed (sst_5_TMD2, sst_5_TMD4, sst_5_TMD1) (Cordoba-Chacon et al., 2010) indicative of a prominent role of truncated sst_5_ variants in this brain area. The distinct expression pattern is important in the context of pharmacological characteristics of truncated variants. Indeed, *in vitro* studies showed that cells expressing the sst_5_TMD2 mainly respond to cortistatin, whereas those expressing sst_5_TMD4 were exclusively activated by somatostatin and those bearing the sst_5_TMD1 responded to both ligands (Cordoba-Chacon et al., 2010, 2011). It is to note that species differences exist since in humans, cortistatin activates sst_5_TMD4, while somatostatin activates the sst_5_TMD5 (Duran-Prado et al., 2009; Cordoba-Chacon et al., 2011).

Somatostatin in the brain exerts a wide variety of physiological functions besides the initially described inhibitory effect on growth hormone release. Its actions include increased locomotor activity (Viollet et al., 2008), memory and learning (Vecsei and Widerlov, 1988; Gastambide et al., 2009), and sleep (Steiger, 2007; Xu et al., 2015), as well as changes in autonomic cardiovascular and gastric functions (e.g., sympatho-inhibitory effect with lowering of heart rate and blood pressure, stimulation of gastric secretion and transit) (Brown and Taché, 1981; Martinez et al., 2000; Bou Farah et al., 2016), immune functions (Gonzalez-Rey et al., 2015) and ingestive behaviors (e.g., increased feeding and drinking; Stengel et al., 2015). Of importance in relation with stress, injection of somatostatin into the brain influences emotional processes exerting anxiolytic and anti-depressant effects (Engin and Treit, 2009; Scheich et al., 2016). However, in contrast to other anxiolytics such as benzodiazepines, somatostatin exerts pro-cognitive effects under healthy (Liguz-Lecznar et al., 2016) and Alzheimer's disease conditions (Epelbaum et al., 2009).

## Response to stress

### Activation of CRF signaling

CRF expression is upregulated in the hypothalamus and the peptide released into the median eminence under conditions of stress leading to pituitary ACTH and subsequently adrenal glucocorticoid hormone release (cortisol in humans and corticosterone in rodents) (Turnbull and Rivier, 1997; Smith and Vale, 2006; Kageyama and Suda, 2009). Moreover, hypothalamic CRF_1_ receptors are also upregulated in rodents exposed to acute or chronic stress by a CRF feed-forward mechanism (Bonaz and Rivest, 1998; Imaki et al., 2001; Konishi et al., 2003; Wan et al., 2014; Eraslan et al., 2015). The use of selective CRF receptor subtype agonists and antagonists as well as transgenic animal models established the primary role of CRF_1_ receptor in driving the stress-related HPA, behavioral, autonomic and visceral responses (Turnbull and Rivier, 1997; Luckey et al., 2003b; Farrokhi et al., 2007; Kehne and Cain, 2010; Taché, 2015).

### Activation of somatostatin signaling

Somatostatin signaling is also activated by different stressors. In particular, immobilization (Negro-Vilar and Saavedra, 1980; Arancibia et al., 2000), handling (Arancibia et al., 1984), maternal separation (Polkowska and Wankowska, 2010), hypoxia (Chen and Du, 2002), pain (Arancibia et al., 1984), and injection of endotoxin (Priego et al., 2005) increase hypothalamic somatostatin mRNA levels and peptide release. Somatostatin release also occurred in the dorsal dentate gyrus in rats subjected to immobilization (Arancibia et al., 2001). Moreover, rats exposed to an elevated plus maze displayed an activation of somatostatin positive neurons in the basolateral amygdala (Butler et al., 2012). The activation of somatostatin signaling under conditions of stress is not restricted to the ligand but also occurred at the receptor level. In the amygdala and anterior cingulate cortex, sst_2_ mRNA was upregulated following acute exposure of rats to a potential predator (Nanda et al., 2008), a finding recently also observed in the medial habenula following chronic mild stress (Faron-Gorecka et al., 2016).

## Suppression of the CRF-mediated stress response by activation of somatostatin signaling

### Endocrine response

Initial reports by Brown et al. showed that intracerebroventricular (icv) injection of the pan-sst agonist, somatostatin-28 or the oligo-somatostatin agonist, des-AA1,2,4,5,12,13-[D-Try_8_]somatostatin (ODT8-SST) (Erchegyi et al., 2008) prevented the increase of ACTH plasma levels induced by tail suspension or exposure to ether in rats (Brown et al., 1984). By contrast, the intravenous (iv) injection of ODT8-SST had no effect indicating a centrally-mediated inhibitory action of somatostatin-28 (Brown et al., 1984). The somatostatin action may involve a component upstream of CRF signaling. This is most likely due to the inhibition of hypothalamic release of CRF induced by tail suspension (Brown et al., 1984). This is also supported by *in vitro* studies showing that octreotide (sst_5_ = sst_2_ > sst_3_ agonist) (Grace et al., 2008) and the pan-sst agonists, somatostatin-14 and cortistatin (sst_2_ = sst_3_ = sst_4_ = sst_5_ > sst_1_) (Fukusumi et al., 1997) blunt basal and KCl-induced CRF release from hypothalamic and hippocampal explants (Tizabi and Calogero, 1992; Tringali et al., 2012).

With regards to the receptor subtype(s) involved, in addition to the sst_5_ = sst_2_ > sst_3_ agonist, octreotide (Tringali et al., 2012), recent studies indicate that intra-hippocampal infusion of the sst_2_ agonist, L-054,264, and sst_4_ agonist, L-803,087 lowered the elevated corticosterone levels in the plasma and hippocampal dialysate induced by acute foot shock in mice (Prévôt et al., 2017). By contrast, under the same conditions, the sst_1_ agonist L-797,591 and sst_3_ agonist, L-796,77 had no effect (Prévôt et al., 2017). The activation of these somatostin-sst_2_ and -sst_4_ signaling pathways was shown to have physiological relevance in dampening the hippocampal corticosterone elevation induced by acute foot shock. In sst_2_ knockout mice, the rise in hippocampal corticosterone after an acute foot shock had a shorter onset, higher maximum and delayed recovery compared to wild type mice (Prévôt et al., 2017). Moreover, the sst_4_ agonist microinjected into the hippocampus in sst_2_ knockout mice exposed to foot shock shortened the return to basal corticosterone levels, while not influencing the elevation to the peak rise (Prévôt et al., 2017). These data point toward distinct inhibitory actions of sst subtypes with sst_2_ dampening the initial stress-corticosterone response, while sst_4_ activation accelerates the recovery.

**Figure 1:**
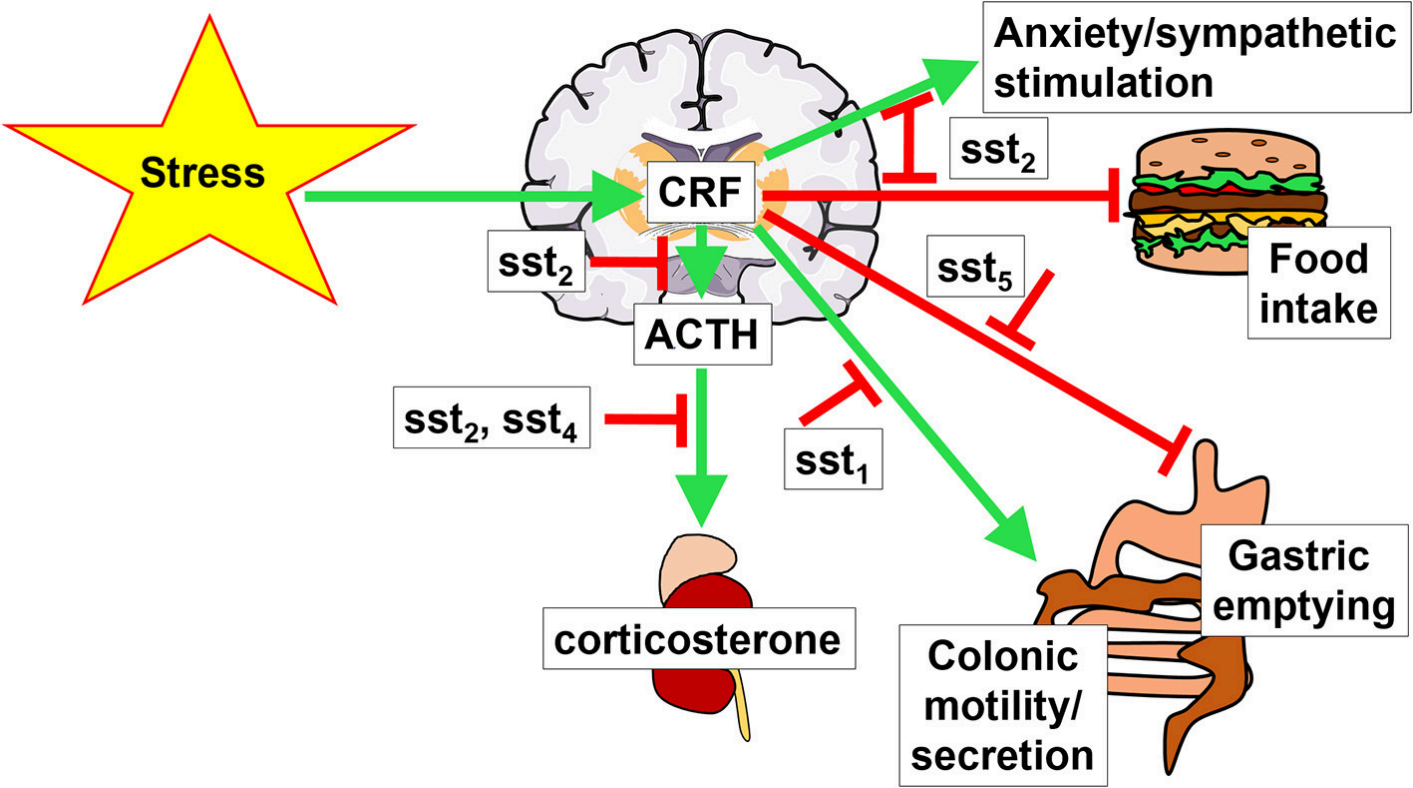
**Brain interaction of CRF and somatostatin signaling**. Stress activates the hypothalamus-pituitary-adrenal gland axis by stimulating the hormones corticotropin-releasing factor (CRF), adrenocorticotropic hormone (ACTH) and corticosterone. This stimulation is modulated by somatostatin signaling *via* different somatostatin receptors (sst). Green arrows indicate a stimulatory effect, red arrows depict an inhibition.

It is likely that somatostatin may have an additional sst_2_-mediated inhibitory action at the pituitary level where ACTH-secreting cells express the sst_2_ (Day et al., 1995; Mezey et al., 1998). *In vitro* studies showed that somatostatin-14 (Heisler et al., 1982; Richardson, 1983) and somatostatin-28 (Strowski et al., 2002) as well as selective sst_2_ and sst_5_ agonists, unlike sst_1_, sst_3_, or sst_4_ (Strowski et al., 2002) block the CRF-stimulated ACTH release from pituitary AtT-20 cells. Moreover, the pituitaries of sst_2_ knockout mice display a higher ACTH release *in vitro* compared to that of wild type littermates (Viollet et al., 2000) and *in vivo*, sst_2_ knockout mice have elevated basal levels of plasma corticosterone, while those of sst_4_ knockout mice were unchanged (Prévôt et al., 2017). Taken together, the somatostatin receptors-induced reduction of HPA activation and elevation of hippocampal corticosterone in response to stress may be primarily mediated by brain sst_2_ and sst_4_ and pituitary sst_2_ (Figure 1).

### Autonomic response

Convergent reports in rats indicate that the activation of brain somatostatin signaling blocked the stress-related sympathetic activation. Brown et al. initially showed that icv injection of somatostatin-28 or ODT8-SST abolished the plasma epinephrine elevation elicited by a variety of stressors including acute exposure to tail suspension, intermittent loud noise, ether stress, or metabolic hypoglycemic stress induced by the injection of 2-deoxyglucose or insulin in rats (Fisher and Brown, 1980; Brown et al., 1982, 1984; Gotoh et al., 2001).

The centrally mediated action of the SST-agonists was demonstrated by the lack of effect when ODT8-SST was administered peripherally at a dose 100-times higher than icv (Fisher and Brown, 1980; Gotoh et al., 2001). Microinjection of ODT8-SST into specific brain nuclei further established that the dorsal hypothalamic area is a brain site responsive to suppress elevated epinephrine secretion in dogs (Brown, 1983). In addition, the direct electrophysiological recording in the adrenal branch of the splanchnic nerve corroborated that icv injection of somatostatin suppresses adrenal sympathetic efferent activity in rats (Somiya and Tonoue, 1984).

With regards to the somatostatin receptor subtype(s) involved, icv injection of octreotide (sst_5_ = sst_2_ > sst_3_) prevented the rise in serum catecholamines induced by 2-deoxyglucose and short exposure to cold swim stress (Gotoh et al., 2001). Recent neuroanatomical and electrophysiological findings support a role of the sst_2_ to induce sympathoinhibitory actions in presympathetic neurons located in the rostroventrolateral medulla (RVLM). The sst_2a_ is the receptor most abundantly expressed at this site compared to other subtypes and microinjection of somatostatin into the RVLM induces a sympatho-inhibitory response mimicked by the sst_2_ agonist, lanreotide and prevented by an sst_2_ antagonist, BIM-23627 in rats (Burke et al., 2008).

### Behavioral response—focus on food intake

Brain CRF receptors are involved in the stress-related reduction of food intake in rodents (Krahn et al., 1986; Shibasaki et al., 1988b) through the activation of both CRF_1_ and CRF_2_ (Hotta et al., 1999; Sekino et al., 2004; Stengel and Taché, 2014b). This inhibitory effect is counteracted by brain somatostatin as the pan-sst agonists, somatostatin-14 and somatostatin-28 or the oligo-somatostatin agonist, octreotide injected icv blunted the icv CRF-induced reduction of refeeding after a fast in rats (Shibasaki et al., 1988a). Moreover, somatostatin-14 and octreotide also blocked the robust anorexigenic response to restraint stress (Shibasaki et al., 1988b). Likewise, we reported that the intracisternal (ic) injection of ODT8-SST prevented the inhibition of food intake induced after abdominal surgery in fasted rats (Stengel et al., 2011b). This effect is recapitulated by the selective peptide sst_2_ agonist, S-346-011 (Stengel et al., 2011b; Figure 1). We also found that ic ODT8-SST or an sst_2_ agonist, unlike selective sst_1_ or sst_4_ agonists, restored plasma levels of the orexigenic hormone, acyl ghrelin (Hosoda et al., 2002) that were inhibited by abdominal surgery (Stengel et al., 2011b). However, the restoration of food intake after surgery by ic ODT8-SST is not secondary to the normalization of circulating acyl ghrelin as the ghrelin receptor antagonist, [D-Lys^3^]-GHRP-6 injected intraperitoneally (ip) did not alter ic ODT8-SST's action (Stengel et al., 2011b).

We previously established that abdominal surgery activates hypothalamic CRF neurons (Bonaz and Taché, 1997; Wang et al., 2011). Therefore, it may be speculated that the activation of brain sst_2_ by ic ODT8-SST suppresses brain CRF release and the related inhibition of food intake (Stengel and Taché, 2014b). Whether the recently established robust dipsogenic response to brain sst_2_ activation (Karasawa et al., 2014) also contributes to the increased feeding behavior (Kissileff, 1969) inhibited by stress will have to be further investigated.

### Visceral response—focus on gastrointestinal motor functions

A multitude of stressors (physical, psychological, and immunological) alter gastrointestinal transit resulting in an inhibitory effect in the upper gastrointestinal tract, while stimulating colonic propulsive motor function (Stengel and Taché, 2009). These effects involve the activation of brain CRF receptors (Taché and Bonaz, 2007). Likewise, CRF and urocortin 1 injected into the brain ventricle or paraventricular nucleus of the hypothalamus suppress gastric emptying (Taché et al., 1987; Mönnikes et al., 1992; Coskun et al., 1997; Lee and Sarna, 1997) and shorten colonic transit time (Mönnikes et al., 1993; Martinez and Taché, 2001). Conversely, the blockade of CRF receptor signaling, namely CRF_2_ and/or CRF_1_ in the upper and CRF_1_ in the lower gastrointestinal tract, prevented the delayed gastric emptying and the stimulation of colonic motility and defecation induced by various stressors in rodents (Taché and Bonaz, 2007).

By contrast to the ic injection of CRF, that of the pan-sst agonist, ODT8-SST accelerates gastric emptying in rats, an effect recapitulated by the preferential sst_5_ agonist, BIM-23052 injected ic and blocked by subdiaphragmatic vagotomy or atropine (Martinez et al., 2000). The sst_5_ is likely the main receptor mediating this action as ic injection of the selective sst_1_, sst_2_, sst_3_ or sst_4_ agonists CH-275, DC-32-87, BIM-23056 and L-803,087, respectively did not modify gastric emptying (Martinez et al., 2000). Lastly, intravenous (iv) injection of the predominant sst_5_ agonist, BIM-23052 had no effect (Martinez et al., 2000). The prominent expression of the sst_5_ in the dorsal motor nucleus of the vagus nerve (Thoss et al., 1995) along with functional data are consistent with the activation of the sst_5_ in the hindbrain inducing a vagal cholinergic-dependent stimulation of gastric propulsive motor function (Martinez et al., 2000). The sst_5_ has been shown to form heterodimers with sst_1_ or sst_2_ that potentiates signaling efficiency (Rocheville et al., 2000). Whether ODT8-SST acts through these heterodimers cannot be ruled out. In addition, as the μ opioid receptor antagonist, naloxone was shown to block the ODT8-SST-induced acceleration of gastric emptying in rats (Stengel et al., 2010), it will be important to investigate whether heterodimers of the sst_5_ with the μ opioid receptor also expressed in the dorsal motor nucleus (Mansour et al., 1995) occur as shown before with the sst_2a_ (Pfeiffer et al., 2002).

Under stress conditions, the activation of somatostatin receptors restores the inhibited gastric emptying. Abdominal surgery is a well-established physical stressor suppressing the initial neurogenic phase of postoperative gastric ileus through activation of brain CRF signaling (Luckey et al., 2003a; Stengel and Taché, 2014a). We showed that injection of somatostatin-28 icv and ODT8-SST icv or ic prevented the abdominal surgery-induced delayed gastric emptying (Stengel et al., 2011b). This effect is mimicked by ic injection of the selective sst_5_ agonist, BIM-23052, while under the same conditions, ic injection of sst_1_ (S-406-062), sst_2_ (S-346-011), or sst_4_ (S315-297) peptide agonists had no effect (Stengel et al., 2011b). It is important to note that the prevention of surgery-induced inhibition of the prokinetic hormone acyl ghrelin (De Smet et al., 2009) by ic ODT8-SST primarily involves the sst_2_ (Stengel et al., 2011b). Additionally, we demonstrated that blockade of ghrelin signaling using the ghrelin receptor antagonist, [D-Lys^3^]-GHRP-6 did not modify the ODT8-SST-induced prevention of postoperative gastric ileus (Stengel et al., 2011b). Taken together these data argue against the preventive action of ic ODT8-SST against postoperative gastric ileus being secondary to the normalization of circulating prokinetic hormone acyl ghrelin. The mechanisms may involve stimulation of vagal efferent activity and/or an interaction with other transmitters such as opioids that will have to be further established.

Several stressors exert a brain CRF_1_-mediated stimulatory action on colonic motor functions in rodents (Taché and Million, 2015). The activation of brain somatostatin signaling inhibits the colonic response to exogenously administered CRF or CRF endogenously released by stress (Stengel et al., 2011a). We reported that icv injection of ODT8-SST inhibits the icv CRF- and water avoidance stress-induced increased fecal pellet output and colonic contractions evoked by semi-restraint in mice (Stengel et al., 2011a). Pharmacological characterization of receptor supports a primary involvement of the sst_1_. Acute anesthesia stress in mice, that led to a pronounced increase of fecal pellet output, is inhibited by icv injection of somatostatin-28 and ODT8-SST or the selective peptide sst_1_ agonist, S-406-062 (Stengel et al., 2011a). In contrast, the oligo-sst agonist, octreotide (sst_5_ = sst_2_ > sst_3_) or selective peptide sst_2_ or sst_4_ agonists, S-346-011 and S-315-297, respectively did not modify the acute stress-induced stimulation of fecal pellet output (Stengel et al., 2011a). This assumption is further corroborated by the expression of this receptor (among other receptors) in the hypothalamus and the brainstem (Fehlmann et al., 2000) in nuclei modulating colonic motility.

## Summary

Mounting evidence supports that the activation of brain sst alleviates many components of the stress response involving brain CRF signaling. The receptor subtype(s) have been characterized by the use of selective peptide agonists and antagonists or genetic manipulations in rodents. The sst_2_ subtype is primarily involved in preventing the acute stress-induced endocrine (rise of ACTH and corticosterone), autonomic (sympatho-inhibition) and behavioral (suppression of food intake and anxiety) responses. With regards to the brain-gut axis, the hindbrain sst_5_ plays a key role in counteracting the stress-induced suppression of gastric emptying, whereas forebrain sst_1_ reduces the stress-related stimulation of propulsive colonic motor function (Figure 1). These data provide clear evidence that exogenous activation of brain sst receptors by pharmacological administration of somatostatin and selective agonists have anti-stress properties; however, the role of endogenous brain somatostatin released by stress (Arancibia et al., 1984, 2000) or exogenous CRF (Mitsugi et al., 1990) in attenuating or terminating the stress response has still been little investigated (Prévôt et al., 2017). Likewise, our knowledge of specific brain sites through which selective activation of somatostatin receptors alleviates the stress response is still limited to sst_2_ signaling in the hippocampus to suppress the rise in plasma corticosterone and to induce anxiolytic behavior (Prévôt et al., 2017) and in the RVLM to elicit sympatho-inhibition (Pilowsky et al., 2008; Prévôt et al., 2017).

## Author contributions

All authors listed, have made substantial, direct and intellectual contribution to the work, and approved it for publication.

### Conflict of interest statement

The authors declare that the research was conducted in the absence of any commercial or financial relationships that could be construed as a potential conflict of interest.
